# Work-related floors as injury hazards – a nationwide pilot project analyzing floors in theatres and education establishments in Germany

**DOI:** 10.1186/s12995-017-0160-y

**Published:** 2017-06-07

**Authors:** Eileen M. Wanke, Mike Schmidt, Doris Klingelhöfer, Jeremy Leslie-Spinks, Daniela Ohlendorf, David A. Groneberg

**Affiliations:** 1 Institute of Occupational Medicine, Social Medicine and Environmental Medicine, Goethe-University, Theodor-Stern-Kai 7, 60590 Frankfurt am Main, Germany; 2 Department of Sports and Exercise Medicine, Institute of Human Movement Science University of Hamburg, Mollerstraße 10, 20148 Hamburg, Germany; 3 School of Performing Arts, University of Wolverhampton, Gorway Rd, Walsall, West Midlands WS1 3BD England

**Keywords:** Work related risks, Dance, Floor, Theatres, Injury prevention

## Abstract

**Background:**

An adequate dance floor is said to prevent injuries. On the basis of scientific research, numerous recommendations regarding an adequate dance floor have been developed. Up to the present, however, studies have still been lacking into how far these recommendations have already been implemented in theatres with regular dance productions and/or in-house dance ensembles. The aim of this study is to analyze a nationwide survey on dance floors of theatres and education establishments in Germany.

**Methods:**

A questionnaire-based survey on existence and type of floors in the various dance-related working areas was carried out at theatres and education establishments institutions (*n* = 86 institutions (*n* = 76 theatres, *n* = 10 education establishments). References as to region, size of dance ensembles and dance styles performed were created.

**Results:**

Of all education establishments, 75.3% were equipped with a sprung sub-floor in the ballet studios. In contrast, sprung sub-floors were only found in 29.7% of the working areas, the stage AND ballet studios in theatres. The percentage of theatres providing sprung sub-floors in all rooms used by dancers is even lower. Considering all dance-related work areas, larger ensembles (>30 dancers) were offered better conditions regarding floors than smaller ensembles (*p* > 0.001). No significant tendencies were found regarding regions or dance styles.

**Conclusion:**

Recommendations concerning an appropriate dance floor have only partly been realized. Besides secured finances for reinstallation, further education of responsible officials and artists is essential. However, accrediting dance as own genre in theatres is the indispensable prerequisite.

## Background

A dance floor is a very important element of the working environment in dance. If the floor is in accordance with the existing standards it can contribute to minimize or even better avoid traumatic injuries as well as chronic work- and floor-related health problems of the musculoskeletal system [1–8]. In cases where the floor is defective (e.g. folds in the performance surface) [9] or even inappropriate (e.g. absent/lacking sprung sub-floor), it may be a health hazard and precipitate incapacity for work [10]. While performance surfaces can be significant in the case of traumatic injuries or occupational accidents, respectively, the properties of a sub-floor play an important role in the occurrence of chronic misuse and overload injury [8, 11].

Although a detailed description of the components of an adequate dance floor is not part of this article, it should be mentioned that in principle a dance floor should include a performance surface (preferably one that meets the requirements of the various dance styles performed), a sprung sub-floor and an underlying sub-floor onto which the sprung sub-floor as well as the performance surface are fitted. This concept is based on several national and international recommendations and standards [12], fine tuning, however, should be done in cooperation with all the parties (e.g. technical staff, theatre management) involved (including dancers) [8].

The same is valid for the performance surface which is to be chosen specifically for the dance styles to be performed [8, 10, 12]. Currently, there is a vast variety of offers available (e.g. point elastic, area elastic, combi-elastic, cushioned or not cushioned Vinyl surface). Depending on various requirements**,** these offers should be carefully tested in order to be sure of installing the optimal dance floor^11^.

For reasons of occupational health and safety numerous authors of relevant studies as well as statutory accident insurers recommend an especially purpose-constructed sprung sub-floor meeting all requirements determined by dance style, age and organization for the work-related activities of professional dancers [9, 12, 13]. Furthermore, an – ideally identical - sprung sub-floor should be fitted in all working areas used by dancers [10], because it is an additional challenge for the body to individually adjust to different types of floors. Even if the existing floors are basically appropriate it would take time to adjust to another floor and in reality there is no time [6]. The same applies to different floor types in one working area such as a basket system with varying area deflection^6^.

### Objective of the present study

On the one hand, the number of studies dealing with the effects of different types of floors on the musculoskeletal system of dancers has considerably increased. On the other hand, published data on the status quo scenario at theatres with regular dance production are still lacking.

Similar to a pilot study, this is the first analysis of dance floors at theatres in Germany. The primary focus was on the sub-floor costing far more and requiring more handling than the performance surface. It was the aim to find out whether, to what extent and by what means (types of floors) the existing recommendations on injury prevention, mis- or overload concerning floors in dance have already been realized [8, 11, 13, 14]. A relative assessment based on region, size of dance ensemble and dance style was created.

## Methods

In the theatre season 2016/17 (August–June/July) a survey was conducted in all theatres in Germany.

Inclusion criteria were as follows:

1. Theatres with own dance company
2. Theatres with scheduled dance performances
3. (University-) Educational establishments with a dance department

A total of *n* = 86 institutions (theatre and education establishments) with that criteria were identified and chosen for the survey. Direct contact persons were either the responsible technical staff or the management of each institution. In order to achieve the highest possible response rate and, thus, attain reliable results, this study was beforehand announced at a nationwide meeting with theatre managements attending.

The survey comprised the following floor key aspects:

- Aspect 1: Construction type of sub floor in the training area(s) (ballet studio(s),
- Aspect 2: Construction type of sub floor on stage,
- Aspect 3: Existence of a mobile floor for touring purposes
- Aspect 4: Total number of dancers in the dance genre/ size of dance ensemble
- Aspect 5: Region/federal province
- Aspect 6: Dance style (classical/neo-classical and/or contemporary/modern)

A pre-test was carried out in a total of *n* = 6 theatres of various sizes without dance genre.

The aspects were limited to keep the utilization of the participants in this pilot project as low as possible. Digital and printed versions of the questionnaire were available.

### Data Analysis

Results were calculated using the PASW Statistics software package, version 21.0 and Excel 2010. Predominantly, the evaluation was carried out in the form of frequency analyses. Chi-Square Tests were used at crucial points to evaluate the differences between the groups. The significance level was set at α = 0.05.

The theatres were categorized according the size of their dance company for the analysis. Dance ensembles with up to 30 dancers were categorized as ‘small to medium’, ensembles with more than 30 dancers as ‘big’.

With regard to dance styles a simplified categorization with either classical/neo classical and contemporary focus was chosen.

Questions concerning raked stages were intentionally neglected even when here and there a possible relation between raked floor and incidence of injuries was stated [14–18]. The types of floors were categorized in ‚dance floors’‚ sports floors’ and ‚other floors’ (not dance appropriate).

Working areas comprised ballet studios as locations for daily dance classes and rehearsals, resp., as well as the stage as location for dance performances and preceding stage rehearsals.

The various types of floors were categorized into dance specific sprung sub-floors and other sprung floors (basket, sports floor etc.) [11, 12]. One point worthy of note is that - among others - a (batten) basket construction is also customary for special dance floors. Therefore, in replying to this question, subjects could assign a basket-construction to dance specific floors. In cases, where that was not done it was presumed that the basket floor used in their establishment was not a dance specific one.

## Results

There were regular dance performances and/or an employed dance company as in-house genre at a total of *n* = 76 theatres and other locations during the theatre season 2016/17 in Germany. In addition to that, there were a total of *n* = 10 education establishments for professional dance. The response rate of the survey was 90% (*n* = 9) from the education establishments and 86.7% (*n* = 65) from the theatres. Not to be discounted was the lack of answers of up to 16.7% depending on the question. Due to the fact that there were no stages at the education establishments and only few guest performances just the ballet studios were analyzed.

### Theatres equipped with sprung sub-floors suitable for dancers

#### Ballet studio only

On the whole, 75.3% of all institutions (theatres and education establishments) were equipped with a sprung sub-floor, 9.4% did not dispose of one**,** with 15.3% not furnishing any particulars. With the theatres the results were similar: 73.3% were fitted with a dance appropriate floor, 10.7% did not dispose of one**,** and 16% did not furnish any particulars.

9 in 10 of the education establishments were equipped with a sprung sub-floor with *n* = 1 not furnishing particulars.

#### Stage only

The situation ‘on stage’ was completely different: a mere 30.3% of all stages were equipped with a sprung sub-floor with 57.9 not being equipped with one and 11.8% not furnishing particulars. Educational establishments remained neglected as they were not equipped with a separate stage.

#### Ballet studio AND stage

Less than one in three theatres (29.7) was equipped with a floor suitable for dance in all areas used by dancers in training, rehearsals and performances**,** with 54.1% not being equipped with one and 16.2% not furnishing particulars.

#### Ballet studio, stage AND touring

**Fig. 1:**
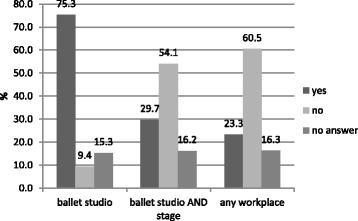
Sprung floors in German theatres with dance productions (*n* = 76)

As even fewer theatres were equipped with a mobile dance floor for touring, the percentage of the institutions equipped with a floor suitable for dance in all working areas decreased to 23.3% compared to 60.5% of the institutions not disposing of a mobile dance floor with 16.3% not furnishing particulars (Fig. 1).

### Type of floors in ballet studios and on stages

Of all institutions equipped with a sprung sub-floor, 33.9% of all ballet studios were equipped with a dance specific sprung sub-floor whereas 66.1%were equipped with another sprung sub-floor (e.g. sports, basket, alternate construction). On the contrary, the stages of the majority of theatres were equipped with a dance (sprung) floor (66.1%) compared to 33.9% equipped with an alternate constructed sprung sub-floor.

### Significance of ensemble size in relation to dance appropriate floors

Big ensembles (> 30 dancers) usually in larger regional capitals or metropolises, respectively**,** have an advantage over smaller ensembles (< 30 dancers). All theatres (100%) with an own dance genre in regional capitals or metropolises were equipped with a dance appropriate floor in the ballet studio compared to a mere 85% of the smaller ensembles (< 30 dancers)(*p* = 0.150).

**Fig. 2:**
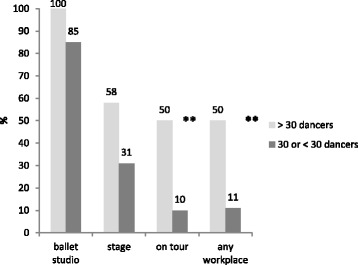
Relative frequency of sprung sub-floors relating to work areas and size of dance companies (*n* = 76) ** highly significant on the 5%- significance level

Of all big ensembles, 58% offered a sprung sub-floor both in the ballet studio and on stage in contrast to only 30% of the smaller ensembles (*p* = 0.073). The differences relating to size of the ensemble and existence of dance appropriate floors in ballet studio, on stage and while touring (*p* = 0.002) (> 30 dancers: 50%, < 30dancers: 11%) is significant. Comparing big and small ensembles with regard to stage there is a tendency (> 30 dancers: 58%, < 30 dancers: 31%, *p* = 0.073) (Fig.2).

### Significance of dance style in relation to dance appropriate floors

Of all contemporary company**,** 89% and 85% of the classical and neo-classical ensembles were equipped with a dance appropriate floor in the ballet studio (*p* = 0.610). Generally speaking, as a tendency more classical and neo-classical ensembles (44%) than contemporary ensembles (33%) were furnished with appropriate floorings on stage (*p* = 0.390). In the ballet studio and on stage, an appropriate dance floor is provided for 36% of the contemporary companies and 42% of the classical and neo-classical companies (*p* = 0.619). Nevertheless, the percentage of appropriate flooring in all working areas was with 27% (classical or neo-classical companies) compared to 14% (contemporary companies almost twice as high, however, as described above, still very low (*p* = 0.249). Hence, ensembles with classical and neo-classical focus were generally speaking – not significantly – better equipped than dance ensembles with contemporary focus.

### Regional significance in relation to dance appropriate floors (East-West Germany)

**Fig. 3:**
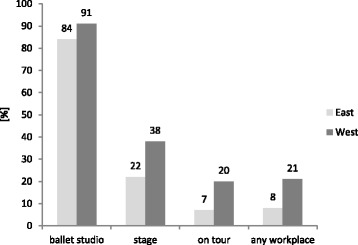
Relative frequency of sprung sub-floors at institutions relating to location (*n* = 76) (Eastern Germany and Western Germany)

There were not significantly more sprung sub-floors in theatres of West Germany compared to East Germany (the former German Democratic Republic), even when the percentage of theatres with sprung sub-floors was slightly higher (Fig.3).

### Regional significance in relation to dance appropriate floors (East-West-North-South)

#### Ballet studio only

**Fig. 4:**
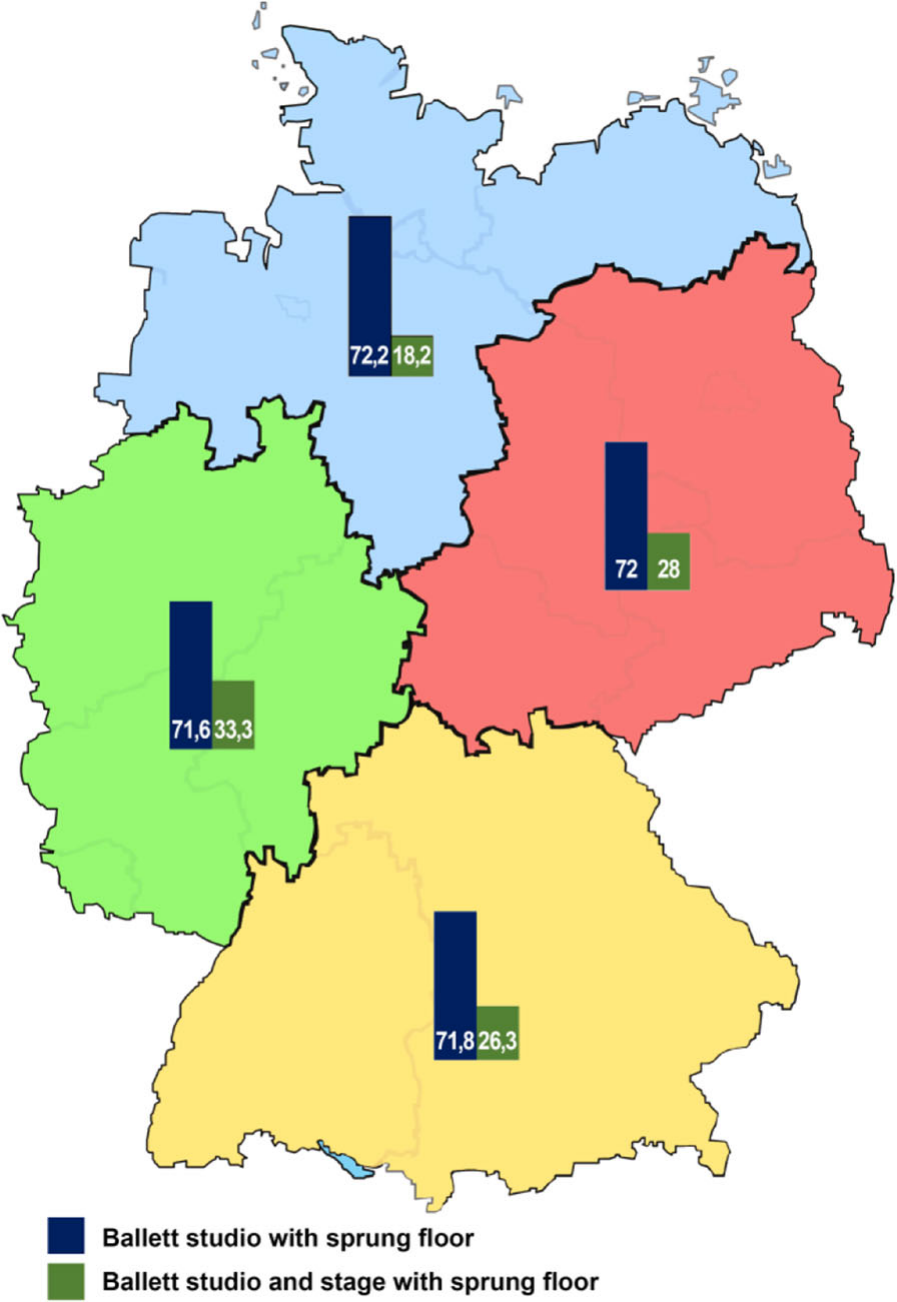
Regional proportional distribution of theatres with sprung sub-floors in ballet studios and ballet studios and on stage (*n* = 76)

With an average of 22.7% not furnishing any particulars and strong regional deviations (East: 12%, West: 27%, North: 27.8%, South: 24%), a comparable percentage of theatres was equipped with sprung sub-floors in ballet studios (Fig.4).

#### Ballet studio and stage

17.6% of all institutions did not furnish any particulars. Significant regional deviations were found with respect to provision of a sprung sub-floor on stage (Fig.4)

## Discussion

The occupational activities of a dancer are accompanied by maximum loads to the musculoskeletal system. Those are repetitive movements, forced postures, slightly limited movements of joints, spine as well as static stop- and support activities or carrying loads (in this case, the dance partner). Over the past 20 years the loads have significantly increased [18]. There is hardly any technical support. As the loads directly affect the dancers’ body a healthy body is essential for the profession. Even minor deficits in the musculoskeletal system can hardly be compensated. A healthy working environment is therefore all the more important in terms of primary preventive measures.

Dance or sports floors are not simply a work tool in dance or other types of sports but also an important contribution towards injury prevention [19, 20]. From a quality point of view, even standardized (after DIN or EN) sports floors are not all equally appropriate for the dance compared to special dance floors due to the existing requirements to a floor and the characteristics of dance (e.g. lack of cushioned footwear) and the anthropometry of female dancers (< 70 kg/11 stones).

The objective of this pilot project was to analyze the present equipment of dance appropriate floors at German theatres by a nationwide survey.

While on the one hand a high percentage of training areas (ballet studios) are equipped with appropriate floors there is on the other hand a great need for this type of flooring on stages and for touring purposes. Fewer than one in four theatres offering regular dance performances are equipped with a dance appropriate floor in all areas and for touring purposes; this results in very variable conditions within the theatre as a work place. Furthermore, the results show that due to the inappropriate floors found in smaller theatres, working conditions are worse for dance ensembles than in bigger theatres (>30 dancers). Concerning dance style and region within Germany just tendencies without any significant value were found.

As a whole, the results show that recommendations in terms of dance floors have only roughly been realized in the ballet studio but not – as recommended - in all other working areas for dancers. Of all theatres surveyed, 75% are not equipped with floors in accordance with the existing recommendations despite their regular dance productions or even own dance companies. The reasons seem to be versatile.

A good sub floor is expensive and long lasting. The first aspect may be the reason why it is the bigger theatres (with big ensembles) in mostly bigger cities and municipalities which have initiated installation of appropriate dance floors. Bigger theatres simply dispose of more financial resources than smaller ones.

The second aspect makes clear that long-lasting floors are much older in relation to the relatively recent research findings and have not yet been replaced because it was not necessary.

The extent to which a lack of information on possible physical effects on working dancers constitutes the reason why so few theatres dispose of dance appropriate floors is yet to be determined. However, the significance of a sprung sub- floor for other theatre genres (drama/opera) is definitely underestimated. As for portable sprung sub-floors**,** it cannot be excluded that particularly the technical management would like to avoid shifting the physical load from dancers to stagehands in cases where a mobile sprung sub-floor consisting partly of heavy panels for dance productions was installed.

Inappropriate floors may be significantly responsible for chronic misuse and overuse complaints sustained by dancers. It is conceivable that these chronic complaints appear years later far beyond any contractual connection. Regarding the causal chain between floor and chronic diseases no research findings have been presented to date. A primary preventive approach towards a dance appropriate floor in all working areas is therefore all the more important.

Hitherto, there seem to have been many – by and large inadequately– explanatory approaches as to why the recommendations relating to appropriate dance floors have been realized in ballet studios but not on stage and or for touring purposes. Further research seems to be inevitable here.

## Conclusion

Recommendations concerning an appropriate dance floor have only partly been realized. Besides secured finances for reinstallation, further education of responsible officials and artists is essential. However, accrediting dance as own genre in theatres is the indispensable prerequisite.
